# Comparison of hemodynamic effects of lidocaine, prilocaine and mepivacaine solutions without vasoconstrictor in hypertensive patients

**DOI:** 10.1590/S1678-77572010000400006

**Published:** 2010

**Authors:** Bahadir EZMEK, Ahmet ARSLAN, Cagri DELILBASI, Kemal SENCIFT

**Affiliations:** 1 DDS, MSc, Department of Oral and Maxillofacial Surgery, Faculty of Dentistry, Yeditepe University, Istanbul, Turkey.; 2 DDS, PhD, Assistant Professor, Department of Oral and Maxillofacial Surgery, Faculty of Dentistry, Yeditepe University, Istanbul, Turkey.; 3 DDS, PhD, Associate Professor, Department of Oral and Maxillofacial Surgery, Faculty of Dentistry, Yeditepe University, Istanbul, Turkey.; 4 DDS, PhD, Professor, Department of Oral and Maxillofacial Surgery, Faculty of Dentistry, Yeditepe University, Istanbul, Turkey.

**Keywords:** Hypertension, Vasoconstrictor agents, Hemodynamics, Anesthesia, Tooth extraction

## Abstract

**Objective:**

Local anesthetic solutions with vasoconstrictors are not contraindicated in hypertensive patients, but due to their hemodynamic effects, local anesthetics without vasoconstrictors are mainly preferred by the clinicians. The aim of this study was to compare hemodynamic effects of three different local anesthetics without vasoconstrictors during tooth extraction in hypertensive patients.

**Material and Methods:**

Sixty-five mandibular molars and premolars were extracted in 60 hypertensive patients (29 females and 31 males; mean age: 66.95 ± 10.87 years; range: 38 to 86 years old). Inferior alveolar and buccal nerve blocks were performed with 2% lidocaine hydrochloride (HCl), 2% prilocaine HCl or 3% mepivacaine HCl without vasoconstrictor. Hemodynamic parameters namely systolic blood pressure (SBP), diastolic blood pressure (DBP), mean arterial pressure (MAP), heart rate (HR), saturation rate (SR), rate pressure product (RPP) and pressure rate quotient (PRQ) were investigated before and at different intervals after anesthetic injection.

**Results:**

The hemodynamic effects of the three agents were similar to each other, although some significance was observed for DBP, MAP, RPP and PRQ values in the lidocaine, prilocaine and mepivacaine groups.

**Conclusion:**

Lidocaine, prilocaine and mepivacaine solutions without vasoconstrictor can be safely used in hypertensive patients. It is advisable that dental practitioners select anesthetic solutions for hypertensive patients considering their cardiovascular effects in order to provide patient comfort and safety.

## INTRODUCTION

Hypertension is the most common chronic systemic disease in adults, and its prevalence tends to rise with age. As the number of hypertensive patients increases, it is expected to encounter more of these patients in dental practice^29^. Local anesthesia is an important concern in these patients as the anesthetic solution may cause serious complications.

Vasoconstrictors present many advantages and can be safely used for most patients treated by dentists. However, the benefits they provide are sometimes outweighed by potential risks of serious medical complications, especially in patients with cardiovascular disorders or other systemic conditions^20,28^. Life-threatening complications due to the sudden and dramatic increase in blood pressure (BP) can occur during dental procedures in hypertensive patients. Although it is stated in the literature that local anesthetics with vasoconstrictors can be safely used during oral surgery in hypertensive patients^21^, there are still some controversies about this subject^12,14^. It has been reported that the use of anesthetic solutions without vasoconstrictors increase the risk of hypertensive crisis due to the potential pain caused by insufficient intraoperative anesthesia^24,25^. Most clinicians prefer using local anesthetics without vasoconstrictors in hypertensive patients due to the negative effects of vasoconstrictors on cardiovascular system. Therefore, hemodynamic aspects, like BP or heart rate (HR), in hypertensive patients come into prominence.

In addition to HR and BP, myocardial ischemia is also important in hypertensive patients. Rate pressure product (RPP) and pressure rate quotient (PRQ) are described as the possible predictors of myocardial oxygen consumption and subsequent ischemia. RPP is defined as the product of systolic BP (SBP) and the HR, and PRQ is defined as the mean arterial pressure (MAP) divided by the HR^8,11,19^. Significant values suggested for RPP range from 12,000 to 20,000 to indicate ischemia and over 20,000 to indicate angina pectoris. It must be noted that 75% of all episodes of myocardial ischemia is silent and develops without anginal symptoms. For this reason, a RPP of 12,000 seems to provide a reasonable target value when monitoring ischemia. The target value for PRQ has been determined to be less than 1.0^8^. Previous studies have searched the hemodynamic effects of vasoconstrictor containing local anesthetic solutions^1,4,12^; and such studies for plain solutions are also few^6,9,17,26^. Therefore, the purpose of this study was to compare the hemodynamic effects of three different anesthetic solutions without vasoconstrictors during tooth extraction in hypertensive patients.

## MATERIAL AND METHODS

This study was performed in 70 consecutive Turkish hypertensive patients referred to the Department of Oral and Maxillofacial Surgery of the Faculty of Dentistry of Yeditepe University, for mandibular posterior tooth extraction. The normal range of BP was defined as systolic recording less than 140 mmHg and a diastolic recording of less than 90 mmHg. Patients who were diagnosed as hypertensive and under routine control by a medical practitioner were included in the study. In order to decrease effects of antihypertensive drugs on cardiovascular parameters, patients were selected from those who only use Ca^++^ channelblockers and beta-blockers. Participants were given detailed information about the study and informed consent was obtained. The same oral surgeon performed the tooth extractions. Sixty-five teeth were extracted under local anesthesia by blocking inferior alveolar (IAN) and buccal nerves and 1.5 mL solution was used for IAN block and 0.5 mL for buccal nerve block. Anesthesia was achieved either by lidocaine HCl 2% (Jetokain Simplex^®^ ampoule, ADeKA, Istanbul, Turkey) or prilocaine HCl 2% (Citanest^®^ flacon, Astra Zeneca, Istanbul, Turkey) or mepivacaine HCl 3% (Isocain^®^ ampoule, Novocol Pharmaceutical of Canada, Ontario, Canada) without vasoconstrictors. Patients with SBP higher than 160 mmHg and DBP higher than 95 mmHg and with any other current medical problems were excluded from the study.

All patients were monitored at different evaluation periods. SBP, DBP, MAP, HR, pulse oxymeter oxygen saturation (SR), RPP and PRQ were recorded as the initial (baseline) hemodynamic values. Then, patients rest for 20 min and same measurements were repeated 3 min before injection. The local anesthesia was administered by inferior alveolar and buccal nerve blocks. An electrocardiogram monitor (Datex Ohmede Cardiocap 5, Helsinki, Finland), a pulse oxymeter, and a BP cuff were used to monitor cardiac and hemodynamic changes before and after delivery of local anesthetic. RPP was calculated by multiplying HR and SBP. RPQ was obtained by dividing MAP by HR. A standard mathematical equation was used to calculate MAP value [MAP=(2DBP+SBP)/3]^13^. All hemodynamic values were recorded at initial, 3 min before the injection and every 3 min after anesthetic injection up to the 15^th^ min. Follow-up periods were determined according to Knoll-Köhler, et al.^15^ (1989) and Fernieini, et al.^10^ (2001). The monitoring procedures were standardized by keeping the cuff on the patient’s left arm, in supine position. Extraction period was defined as the duration from initiation of the injection to accomplishing the tooth extraction.

Statistical tests were performed using NCSS 2007 program for Windows. The following tests were used: one-way ANOVA for descriptive statistical methods (mean and standard deviation) and for repetitive analysis of multiple groups; Newman Keuls multiple-comparison test for comparison of the subgroups; one-way ANOVA for intergroup comparisons; Tukey’s multiple comparison test for comparison of the subgroups; and chi-square tests for comparison of qualitative data. P values ≤ 0.05 was considered as significant.

## RESULTS

Seventy consecutive hypertension patients were enrolled for the study. Three patients were excluded as additional anesthetic solution was injected due to inadequate anesthesia; four patients were excluded because they declared during the study that they were using other systemic drugs; three patients were excluded because they have excessive dental anxiety. This way, 65 teeth (57 molars and 8 second premolars) were extracted due to periodontal problems, extensive caries or prosthetic reasons from 60 hypertensive patients (29 females and 31 males; mean age: 66.95 ± 10.87 years; range: 38 to 86 years old). Chi-square test was used to investigate the demographic data and no significant differences were found among the groups with respect to age (p=0.069), gender (p=0.935), type of extracted tooth (p=0.597) and extraction period (p= 0.169). Twenty-one teeth were extracted with lidocaine 2% HCl, 22 teeth were extracted with 2% prilocaine HCl and 22 teeth were extracted with 3% mepivacaine HCl (Table 1).

**Tabela 1:** Distribution of the patients, teeth extracted and extraction periods

		**Lidocaine HCl**	**Prilocaine HCl**	**Mepivacaine HCl**		**p**
						
Gender	Male	11	10	10	X^2^: 0.13	0.935
	Female	9	10	10		
Mean age(yrs)	Male	59.88±7.8	67.9±9.24	69±8.09	F:2,95	0.069
	Female	70.27±7.77	59.6±12.86	74±9.7		
	Mean	65.6±9.34	63.75±11.94	71.5±9.28		
Teeth extracted	Molar	20	18	19	X^2^: 2.76	0.597
	Premolar	1	4	3		
Extraction period (Min)		13.91±8.13	10.14±4.22	10.56±4.92	F:1.84	0.169

There was no significant difference (p>0.05) among the three groups and subgroups (lidocaine, prilocaine and mepivacaine groups separately) for the mean SBP values during the observation periods (Table 2). After the delivery of local anesthesia, the SBP increased in the prilocaine and mepivacaine groups, but this increase was not statistically significant (p>0.05). Significant differences were noted for mean DBP change in lidocaine (p=0.025) and prilocaine groups (p=0.016) during the observation periods (Table 2). There was no significant difference (p>0.05) among the three groups in mean MAP values during the observation periods; however, significant differences were noted for the mean MAP change in lidocaine (p=0.013) and prilocaine groups (p=0.001) during the observation periods (Table 2).

**Tabela 2:** Mean systolic blood pressure (SBP), diastolic blood pressure (DBP) and mean arterial pressure (MAP) in the lidocaine, prilocaine and mepivacaine groups. Data are present as mean ± standard deviation

	**Anesthetic Solution**	**Initial**	**3 min before injection**	**3 min after injection**	**6 min after injection**	**9 min after injection**	**12 min after injection**	**15 min after injection**	**p**
									
SBP (mmHg)	Lidocaine HCl	147.65±11.71	147.35±15.02	144.5±15.14	144.15±15.4	145.55±19.24	139.9±13.65	141.65±14.18	0.139
	Prilocaine HCl	145.95±14.93	151±21.13	144.95±21.83	144.75±18.88	145.5±16.94	141.15±19.44	140.25±17.31	0.098
	Mepivacaine HCl	151.3±12.07	154.3±12.4	151.55±14.67	153.55±12.48	152.05±13.3	150.25±14.1	146.9±13.76	0.344
	p	>0.05	>0.05	>0.05	>0.05	>0.05	>0.05	>0.05	
DBP (mmHg)	Lidocaine HCl	83.5±10.23	86.1±11.25	83.1±11.14	84.85±11.95	86.3±11.13	80.65±9.13	82.25±10.56	**0.025**
	Prilocaine HCl	85.3±6.99	89.7±10.67	86.35±10.87	87.95±10.1	85.8±11.33	84.3±11.28	82.2±13.05	**0.016**
	Mepivacaine HCl	86±7.83	86.4±10.58	86.25±10.96	89.15±13.31	88±13.78	86.65±9.84	86.9±11.97	0.758
	p	>0.05	>0.05	>0.05	>0.05	>0.05	>0.05	>0.05	
MAP (mmHg)	Lidocaine HCl	106.65±10.28	108.75±10.79	106.75±12.48	107.25±10.67	106.65±12.03	100.9±10.45	102.55±11.56	**0.013**
	Prilocaine HCl	106.7±11.91	113.6±15.31	110.3±13.95	110.1±13.12	108.3±13.9	105.45±13.84	103.1±14.03	**0.001**
	Mepivacaine HCl	112.15±10.55	114.05±9.69	109.35±10.52	114.1±12.57	112.6±12.98	111.3±10.74	109.8±11.85	0.063
	p	>0.05	>0.05	>0.05	>0.05	>0.05	>0.05	>0.05	

Bold numbers indicate significant changes for intragroup comparison.

Table 3 shows HR and SR measurements. Significant differences were determined in the mean HR during injection, at the 3rd min after injection and at the 12^th^ min after the injection among the three groups (p<0.05). Significant differences were also noted for the mean HR change in the lidocaine (p=0.019) and mepivacaine groups (p=0.004) during the observation periods (Table 3). There was no significant differences (p>0.05) among the three groups and subgroups for the mean SR values during observation periods (Table 3).

**Tabela 3:** Mean heart rate (HR) and saturation sate (SR) in the lidocaine, prilocaine and mepivacaine groups

	**Anesthetic Solution**	**Initial**	**3 min before injection**	**3 min after injection**	**6 min after injection**	**9 min after injection**	**12 min after injection**	**15 min after injection**	**P**
									
HR(Beat/min)	Lidocaine HCl	83.8±11.51	82.7±10.32	80.75±11.92	80.45±10.08	80.4±10.62	81.65±11.69	80.25±9.14	**0.019**
	Prilocaine HCl	84.3±14.12	82.6±13.87	83.7±14.19	82±11.7	81.9±13.3	81.75±12.66	80.85±14.47	0.435
	Mepivacaine HCl	76.75±12.68	74.75±13.74	72.7±12.42	74.35±13.14	72.8±14.35	72.05±13.62	72±13.96	**0.004**
	p	>0.05	**0.039**	**0.026**	>0.05	>0.05	**0.027**	>0.05	
SR (%)	Lidocaine HCl	97±2.27	96.45±1.98	96.55±1.79	95.9±1.83	96.35±1.98	95.4±2.87	95.65±1.78	0.231
	Prilocaine HCl	96.65±1.81	97±2.15	96.8±1.98	96.15±2.08	95.6±4.81	96.25±1.74	95.6±2.37	0.308
	Mepivacaine HCl	96.1±2.59	96.15±1.87	96.2±1.93	95.7±2.12	96.2±2.83	96.05±1.63	95.9±1.8	0.957
	p	>0.05	>0.05	>0.05	>0.05	>0.05	>0.05	>0.05	

Bold numbers indicate significant changes for intragroup comparison.

Table 4 shows RPP and PRQ measurements. For RPP, 63/140 (45%) measurements in the lidocaine group, 67/140 (47.8%) measurements in the prilocaine group and 55/140 (39.2%) measurements in the mepivacaine group ranged from 12,000 to 20,000. There were no significant differences (p>0.05) among the three groups for the mean RPP values during the observation periods; however significant changes were noted in the lidocaine (p=0.017) and prilocaine groups (p=0.049) during the observation periods (Table 4). For PRQ, 6/140 (4.2%) measurements were <1 the in lidocaine group and 10/140 (7.1%) measurements were <1 in the prilocaine group. All PRQ measurements (100%) in the mepivacaine group were ≥1.

**Tabela 4:** Mean RPP and PRQ in the lidocaine, prilocaine and mepivacaine groups

	**Anesthetic Solution**	**Initial**	**3 min before injection**	**3 min after injection**	**6 min after injection**	**9 min after injection**	**12 min after injection**	**15 min after injection**	**P**
									
RPP	Lidocaine HCl	12315.8±2224.75	12259.55±2214.23	11645.3±1982.12	11596.4±1923.24	11724.95±2394.75	11324.9±1841.21	11314.85±1908.88	**0.017**
	Prilocaine HCl	12336.05±2614.1	12489.1±3081.66	12331.75±2754.09	11980.1±2477.59	11981.55±2716.08	11568.95±2672.47	11357.05±2676.52	**0.049**
	Mepivacaine HCl	11736.05±2510.84	11427.45±2622.49	11071.75±2426.41	11687.05±2628.97	11247.75±2731.14	11220.75±2760.02	10953±2872.82	0.145
	P	>0.05	>0.05	>0.05	>0.05	>0.05	>0.05	>0.05	
PRQ	Lidocaine HCl	1.29±0.18	1.33±0.2	1.35±0.28	1.35±0.2	1.34±0.22	1.25±0.23	1.3±0.22	**0.029**
	Prilocaine HCl	1.35±0.3	1.41±0.31	1.35±0.27	1.47±0.32	1.44±0.31	1.31±0.24	1.32±0.29	**0.013**
	Mepivacaine HCl	1.48±0.21	1.6±0.32	1.54±0.29	1.58±0.36	1.6±0.37	1.59±0.31	1.57±0.3	0.143
	**P**	**0,033**	**0.011**	**0.048**	**0.049**	**0.032**	**0.0001**	**0.004**	

Bold numbers indicate significant changes for intragroup comparison.

Significant differences (p<0.05) were found among the three groups at baseline (initial), 3 min before the injection, and 3, 6, 9, 12 and 15 min after the injection for the mean PRQ values. Significant changes were also noted in the lidocaine (p=0.029) and prilocaine groups (p=0.013) during the observation periods (Table 4).

The results of the one-way ANOVA, Tukey’s post-hoc multiple-comparison test, repeated oneway ANOVA and Newman Keuls post-hoc multiple comparison tests are shown in Tables 2-4.

## DISCUSSION

Most studies on the hemodynamic effects of local anesthetic solutions were done with vasoconstrictor containing solutions. However, these effects can be due not only to vasoconstrictor but to the agent itself^22^. There are few researches on the effects of plain solutions; therefore this study may help dental practitioners to have some information about the hemodynamic effects of the most commonly used anesthetic solutions for hypertensive patients.

The major concern in dentistry is perioperative hypertension crisis in hypertensive patients. As hypertension can bring about complications such as paralysis, heart and renal problems, and acute medical problems, hypertensive patients constitute an important risk group in dental treatment.

Meiller, et al.^23^ (1983) in a study on normotensive and hypertensive patients, determined that during local anesthesia and tooth extraction that BP increased continually, though without statistical significance. In the present study, SBP did not show statistically significant differences among the groups, but significant changes were observed in the lidocaine and prilocaine groups in DBP and MAP, though not in increasing fashion. This may be due to the agents themselves or to the dental anxiety felt by the patients.

Holm, et al.^14^ (2006) reported hypertensive crisis as SBP of at least 250 mmHg, DBP of at least 130 mmHg, or both. In the present study, none of the patients presented such high SBP or DBP rates, and no cardiovascular complications occurred during tooth extractions.

The lack of a control group in the present study consisting of epinephrine-containing local anesthetic to verify the effect of epinephrine on hemodynamic values in hypertensive patients may be open to criticism. However, as our clinical protocol for hypertensive patients does not include the use of epinephrine-containing anesthetics, it would be unethical to form such a comparison group in this study. In addition, it was not the study purpose to evaluate correlations between hemodynamic parameters and age, gender or extraction periods, which may be investigated in another study.

The present finding that HR decreased significantly in the lidocaine and mepivacaine groups is similar to the findings of earlier studies^27,32^. Paramaesvaran and Kingon^27^ (1994) found that 33 out of the 35 patients who received local infiltrations had a decrease in the HR during or after anesthesia. Yokobayashi, et al.^32^ (1977) found that administration of local anesthesia lowered the HR, and local infiltration resulted in a more significant effect than block anesthesia. Meanwhile Liau, et al.^18^ (2008), reported an increase in the HR and the BP during local anesthesia. Bradycardia due to local anesthesia may be related with the effect of local anesthetic itself. Van Rooij, et al.^30^ (2004) reported that lidocaine may develop bradycardia in neonates. An animal study also demonstrated that lidocaine may decrease the HR^2^ and mepivacaine can also cause bradycardia^3^. It has been reported that abnormal electrocardiography changes may occur during local anesthesia delivery and oral surgery in 15 %-37.5 % of the patients^5,7^. The differences among the studies may have been due to different study populations or to the dose of anesthetic used. Various antihypertensive drugs have different effects on hemodynamic parameters such as vasodilatation, decreasing peripheral resistance, decreasing BP and bradycardia. Therefore, in order to provide standardization, patients using Ca^++^ channel-blockers and beta-blockers only were included in the study.

There were no significant changes in SR rates among the groups. In the literature, it is reported that prilocaine and lidocaine may cause methemoglobinemia^16,31^. Liau, et al.^18^ (2008) stated that SR changes correlated with pain on injection before local anesthesia delivery, but not during the postinjection period, and the extent to this correlation was attributable to factors influencing the perception of pain, such as anxiety and past dental experience. We did not evaluate the stress and dental anxiety secondary to injection and tooth extraction in this study. Further studies are necessary to include the effects of stress and anxiety on the relationship between anesthetic injection and hemodynamic changes.

Myocardial ischemia occurs when the oxygen demands of the myocardium exceeds the supply. Demand is affected by the HR, preload, afterload and contractility. The clinical manifestations are quite variable, with many patients free of symptoms whereas others experience varying degrees of angina pectoris^8^. In the present study, significant differences in RPP and PRQ values were noted between the lidocaine and prilocaine groups. However, clinical signs and symptoms of clinical disturbance or angina attack were observed in any of the patients.

Local anesthesia is commonly used in dental practice. Hypertensive patients constitute a large number among patients with systemic diseases. Selection of a proper anesthetic solution will provide patient safety and satisfaction. Further evaluation of the efficiency and safety of different anesthetic solutions in patients with systemic diseases is advisable.

## CONCLUSION

It may be concluded that the hemodynamic effects of the three local anesthetic solutions evaluated in this study were similar to each other, and they could be safely used during tooth extractions in hypertensive patients, though significant variations may arise probably due to the anesthetic properties.

## References

[1] Alemany-Martinez A, Valmaseda-Castellón E, Berini-Aytés L, Gay-escoda C (2008). Hemodynamic changes during the surgical removal of lower third
molars. J Oral Maxillofac Surg.

[2] Al Rasheed NM, Al Sayed MI, Al Zuhair HH, Al Obaid AR, Fatani AJ (2001). Effects of two newly synthesized analogues of lidocaine on rat
arterial blood pressure and heart rate. Pharmacol Res.

[3] Ayestaran C, Matorras R, Gomez S, Arce D, Rodriguez-escudero F (2000). Severe bradycardia and bradpynea following vaginal oocyte retrieval: a
possible toxic effect of paracervical mepivacaine. Eur J Obstet Gynecol Reprod Biol.

[4] Bader JD, Bonito AJ, Shugars DA (2002). A systematic review of cardiovascular effects of epinephrine on
hypertensive dental patients. Oral Surg Oral Med Oral Pathol Oral Radiol endod.

[5] Blinder D, Shemesh J, Taicher S (1996). Electrocardiographic changes in cardiac patients undergoing dental
extractions under local anesthesia. J Oral Maxillofac Surg.

[6] Cáceres MT, Ludovice AC, Brito FS, Darrieux FC, Neves RS, Scanavacca MI (2008). Effect of local anesthetics with and without vasoconstrictor agent in
patients with ventricular arrhythmias. Arq Bras Cardiol.

[7] Campbell JH, Huizinga PJ, Das SK, Rodriguez JP, Gobetti JP (1996). Incidence and significance of cardiac arrhytmia in geriatric oral
surgery patients. Oral Surg Oral Med Oral Pathol Oral Radiol endod.

[8] Campbell RL, Langston WG, Ross GA (1997). A comparison of cardiac rate-pressure product and pressure-rate
quotient with Holter monitoring in patients with hypertension and cardiovascular
disease: a follow-up report. Oral Surg Oral Med Oral Pathol Oral Radiol endod.

[9] Conrado VC, Andrade J, Angelis GA, Andrade AC, Timerman L, Andrade MM (2007). Cardiovascular effects of local anesthesia with vasoconstrictor during
dental extraction in coronary patients. Arq Bras Cardiol.

[10] Fernieini EM, Bennett JD, Silverman DG, Halaszynski TM (2001). Hemodynamic assessment of local anesthesic administration by laser
Doppler flowmetry. Oral Surg Oral Med Oral Pathol Oral Radiol Endod.

[11] Fornitano LD, Godoy MF (2006). Increased rate pressure product as predictor for the absence of
significant obstructive coronary artery disease in patients with positive exercise
test. Arq Bras Cardiol.

[12] Gungormus M, Buyukkurt MC (2003). The evaluation of the changes in blood pressure and pulse rate of
hypertensive patients during tooth extraction. Acta Med Austriaca.

[13] Haque IU, Zaritsky AL (2007). Analysis of the evidence for the lower limit of systolic and mean
arterial pressure in children. Pediatr Crit Care Med.

[14] Holm SW, Cunningham LL, Bensadoun E, Madsen MJ (2006). Hypertension: classification, pathophysiology and managament during
outpatient sedation and local anesthesia. J Oral Maxillofac Surg.

[15] Knoll-Köhler E, Frie A, Becker J, Ohlendorf D (1989). Changes in plasma epinephrine concentration after dental infiltration
anesthesia with different doses of epinephrine. J Dent Res.

[16] Kreutz RW, Kinni ME (1983). Life threatening toxic methemoglobinemia induced by
prilocaine. Oral Surg Oral Med Oral Pathol.

[17] Laragnoit AB, Neves RS, Neves IL, Vieira JE (2009). Locoregional anesthesia for dental treatment in cardiac patients: a
comparative study of 2% plain lidocaine and 2% lidocaine with epinephrine
(1:100,000). Clinics (Sao Paulo).

[18] Liau FL, Kok SH, Lee JJ, Kuo RC, Hwang CR, Yang PJ (2008). Cardiovascular influence of dental anxiety during local anesthesia for
tooth extraction. Oral Surg Oral Med Oral Pathol Oral Radio Endod.

[19] Lindner O, Vogt J, Kammeier A, Wielepp P, Holzinger J, Baller D (2005). Effect of cardiac resynchronization therapy on global and regional
oxygen consumption and myocardial blood flow in patients with non-ischaemic and
ischaemic cardiomyopathy. Eur Heart J.

[20] Little JW (2000). The impact on dentistry of recent advances in the management of
hypertension. Oral Surg Oral Med Oral Pathol Oral Radiol Endod.

[21] Malamed SF, Malamed SF (2004). Clinical action of specific agents. Handbook of local anaesthesia.

[22] Malamed SF, Malamed SF (2003). Pain and anxiety in dentistry. Sedation: a guide to patient management.

[23] Meiller TF, Overholser CD, Kutcher MJ, Bennett R (1983). Blood pressure fluctuations in hypertensive patients during oral
surgery. J Oral Maxillofac Surg.

[24] Meyer FU (1987). Haemodynamic changes under emotional stress following a minor surgical
procedure under local anaesthesia. Int J Oral Maxillofac Surg.

[25] Meyer FU (1986). Hemodynamic changes of local dental anesthesia in normotensive and
hypertensive subjects. Int J Clin Pharmacol Ther Toxicol.

[26] Neves RS, Neves IL, Giorgi DM, Grupi CJ, César LA, Hueb W (2007). Effects of epinephrine in local dental anesthesia in patients with
coronary artery disease. Arq Bras Cardiol.

[27] Paramaesvaran M, Kingon AM (1994). Alterations in blood pressure and pulse rate in exodontia
patients. Aus Dent J.

[28] Perusse R, Goulet JP, Turcotte JY (1992). Contraindications to vasoconstrictors in dentistry: Part I.
Cardiovascular diseases. Oral Surg Oral Med Oral Pathol.

[29] Sproat C, Beheshti S, Harwood AN, Crossbie D (2009). Should we screen for hypertension in general dental
practice?. Br Dent J.

[30] Van Rooij LG, Toet MC, Rademaker KM, Groenendaal F, de Vries LS (2004). Cardiac arrhytmias in neonates receiving lidocaine as anticolvulsive
treatment. Eur J Pediatr.

[31] Wilburn-Goo D, Lloyd LM (1999). When patients become cyanotic: acquired
methemoglobinemia. J Am Dent Assoc.

[32] Yokobayashi T, Nakajima T, Yagata H, Yatabe Y (1977). Changes of heart rate during administration of local anesthetics in
the oral region. J Oral Surg.

